# Reducing Greenhouse Gas Emissions in Grassland Ecosystems of the Central Lithuania: Multi-Criteria Evaluation on a Basis of the ARAS Method

**DOI:** 10.1100/2012/908384

**Published:** 2012-05-03

**Authors:** Ligita Balezentiene, Albinas Kusta

**Affiliations:** Environment Institute, Aleksandras Stulginskis University, Studentu 11, Akademija, 53361 Kaunas, Lithuania

## Abstract

N_2_O, CH_4_, and CO_2_ are potential greenhouse gas (GHG) contributing to climate change; therefore, solutions have to be sought to reduce their emission from agriculture. This work evaluates GHG emission from grasslands submitted to different mineral fertilizers during vegetation period (June–September) in two experimental sites, namely, seminatural grassland (8 treatments of mineral fertilizers) and cultural pasture (intensively managed) in the Training Farm of the Lithuanian University of Agriculture. Chamber method was applied for evaluation of GHG emissions on the field scale. As a result, soil chemical composition, compactness, temperature, and gravimetric moisture as well as biomass yield of fresh and dry biomass and botanical composition, were assessed during the research. Furthermore, a simulation of multi-criteria assessment of sustainable fertilizers management was carried out on a basis of ARAS method. The multicriteria analysis of different fertilizing regimes was based on a system of environmental and productivity indices. Consequently, agroecosystems of cultural pasture (N_180_P_120_K_150_) and seminatural grassland fertilizing rates N_180_P_120_K_150_ and N_60_P_40_K_50_ were evaluated as the most sustainable alternatives leading to reduction of emissions between biosphere-atmosphere and human-induced biogenic pollution in grassland ecosystems, thus contributing to improvement of countryside environment.

## 1. Introduction

The global mean temperature is expected to increase significantly; hence, there is a growing risk of climate change and concomitant extreme climatic events [1]. United Nations has summarized anthropogenic forcing of climate change due to GHG annual increase of 0.4 (CO_2_), 0.6 (N_2_O), and 0.25% (CH_4_) [2, 3]. Therefore, it is actual to reduce the main driver of climate change, that is, anthropogenic greenhouse gas emissions in agricultural sector as well as in other activities. As Eurostat (2006) reports, the European Union (EU) having 5% global population contributes up to 15% of total GHG emissions [4]. Deeper emissions cuts will be needed after 2012 if the international community is to win the battle against climate change, and further EU policies and measures will be required to achieve these [5]. Consequently, the commission has initiated the Second European Climate Change Programme (ECCP II). The 27 European Union member states committed themselves in 2007 to reduce emissions from 1990 levels by 20% by 2020 [6–8]. 

Agriculture land occupies about 40–50% land surface and generates about 10–12% of the total global anthropogenic emissions, or 5.1–6.1 Gt CO_2_-eq per year. Hence, agricultural sector must take part in mitigating climate change [9–11].

Agriculture contributes to 9% (462.22 Mt CO_2_-eq/yr) of GHG emissions and follows emissions from the energy sector of 27 EU member states [12–14]. Therefore, great attention is paid for cross-cutting measures in agriculture sector. Over 20 measures (Directive 96/61/EC; Landfill Directive 1999/31/EC; Regulations 795/2004/EC, 1655/2000/EC, and 1682/2004/EC; etc.) that include environment-friendly farming and investments to improve farms ecological value and lead to emissions cuts are implemented in the ECCP II policies. By proposing aims for 2007–2013, the commission stressed the need of strengthening the environmental aspect by declaring improvement of the environment and the countryside through land management, one of the main objectives.

Synthetic fertilizers have played an important role in maintaining crops, including grasslands, productivity over the past 50–60 years taking into account the needs of the developed market economies throughout the world and also in Lithuania [15–17]. On the other hand, synthetic fertilizers (also their production) are considered as significant drivers of the development of GHG emissions, whose ultimate outcomes are climate change [18–22]. According to a recent inventory, approximately 75% of anthropogenic N_2_O in Europe is produced by agricultural soils and animal husbandries [8].

Agriculture sector started to raise concerns over the potential overuse and environmental impacts of synthetic fertilizer, especially nitrogen (N), application. Since then, a growing body of research has identified the need to improve fertilizer use efficiencies and management [23]. Application of integrated assessment models is important in fertilizing-benefit analysis to determine the optimal level of GHG emissions mitigation in fertilized grasslands. Grasslands (3488 M ha, or 69%) occupy a large segment of global agricultural land (5023 M ha), and consequently, measurement and prediction of GHG emissions from these ecosystems are of great importance [24]. Furthermore, amount and composition of covering plant species considerably impact total GHG emission in grassland ecosystems [25–27].

Total grassland area in Lithuania occupies 1.2 M ha. In Central Lithuania, like in other parts of central Europe, abandoned grasslands situated near woodlands are overgrown by shrubs and trees [28]. An increase in tree and shrub cover results in a decrease of the number and cover of grassland species and may lead to their local extinction within decades. Domestic political-economical circumstances have meant that about 50% of grasslands (former pasture or arable land) have been abandoned and have been turning into natural habitats of climatic ecosystems during the last two decades in Lithuania [29]. In order to maintain soil fertility and imminent growing up with shrubs and trees, these abandoned, differently anthropogenized plots need to undergo an extensive management, for example, sustainable fertilizing, grazing, and so forth [20, 28]. However, rising fertilizer use contributed to a number of environmental problems including an increase of GHG emissions [30–32]. Moreover, intensive recycling and often high rates of applied mineral fertilizers are expected to be significant pathway for contribution to share of global anthropogenic GHG emission from agrosector [1, 33, 34]. Therefore, assessment of effects of various fertilizing rates and techniques on the gaseous emissions from abandoned grasslands should be based on research data [35]. Otherwise, agroecosystems are represented by complex of multidimensional components, thus making their evaluation and management rather complicated. Hence, their evaluations require appropriate analysis techniques including mathematical methods [36, 37]. In order to address this context, it is necessary to move away from the assessment methods that have traditionally predominated in agroecosystems management. Multi-criteria decision-making (MCDM) methods offer integration of multiple stakeholders interests, thus leading to more robust analysis and relevant policy option to deal with environmental issues [38–41]. Hence, numerous examples of MCDM methods application in ecological sciences are present [42, 43]. Therefore, ARAS method developed by Zavadskas and Turskis [44] will be applied in this study.

This study focuses on integrated assessment of sustainable management of abandoned grasslands aimed at a proper management of fertilizer application methods leading to reduction of GHG emissions, which in turn are significant drivers of air pollution and climate change. The main aim of this investigation was to compare the impact of a single as well as multiple fertilizers on long-living biogenic greenhouse gas (CO_2_, N_2_O, and CH_4_) emissions and to determine the optimal fertilizing schemes in seminatural sward and cultural pasture ecosystems.

In order to improve anthropogenic GHG inventory in agroecosystems and assess the viability of mitigation options, fertilizing risk management and biogenic environment pollution was evaluated applying new additive ratio assessment (ARAS) method. Multi-criteria analysis will contribute to an objective finding of environment-friendly solution.

## 2. Metohds

### 2.1. Study Site

**Figure 1:**
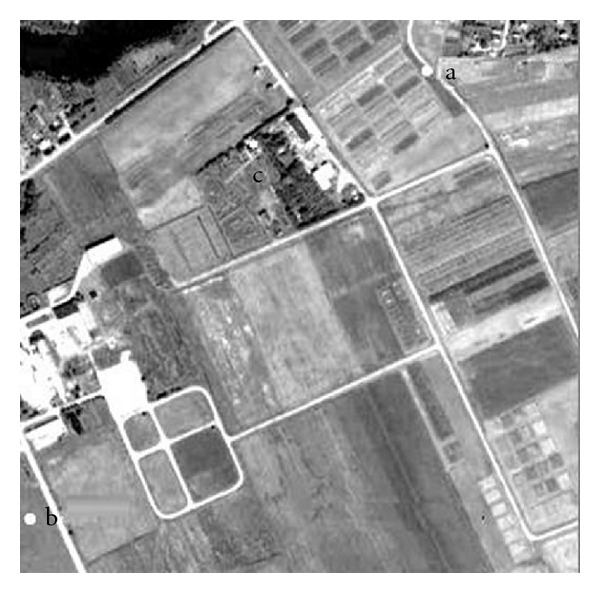
Study sites: (a) seminatural grassland (54°88′N, 23°84′E) and (b) intensively managed cultural pasture (CP); (c) Kaunas Meteorology Station (54°87′N, 23°83′E) (LUA, Research Station; LTD BK 50000-V NŽT, 2004, HNIT-BALTIC GIS, 2005; M 1 : 50000).

The measurements were conducted on two sites: abandoned for more than 20 years grassland (54°88′N, 23°83-84′E) and intensively managed cultural pasture (54°87′N, 23°83′E) have been situated at the Lithuanian University of Agriculture, Kaunas district, during vegetation period of 2009 (Figure 1). The site is located in 5-6 hardiness zone [45] of temperate climate (C) with moderate warm summer and moderate cold winter [46]. Mean annual temperature ranges between 5.5 and 7.5°C with annual precipitation of 670 mm. Total solar radiation inflow amounts 3600 MJ m^−^² in Lithuania. Meteorological data (air temperature and precipitation) are obtained from Kaunas meteorology station, which is situated nearby study site (Figure 1). 

Both the sites of soil was clay loam topsoil over silt loam (*Calc(ar)i-Endohypogleyic Luvisol*) [47]. Humus horizon was 25 cm deep. Soil pH was 6.75–6.97, humus content was 2.48–2.51%, P2O5 was 239–242 mg kg^−1^, and K_2_O was 120–144 mg kg^−1^ in spring. Soil samples were taken at a 15–20 cm depth using auger (2.5 cm in diameter) with 6–8 boreholes per replicated plot, and composite soil samples were formed in accordance with ISO 10 381–1 : 2002. The soil samples were preconditioned for 15–20 days at laboratory temperature (approximately 22°C) before analysis. Soil chemical composition (Table 1) was used for evaluation of correlation with treatment biogenic microgas emissions.

Soil pH was recorded potentiometrically using 1 n KCl extraction, mobile P_2_O_5_ and K_2_O (mg kg^−1^ of soil)—by the Egner-Riehm-Domingo (A–L) method [48]. Soil gravimetric moisture was also continuously recorded using probe (HydroSense Campbell CS-620), and soil bulk density was measured with meter (Fieldscout SC900 Spectrum Technologies) [49].

### 2.2. Experiment Setup

Field test area of each fertilizing treatment was 10 m^2^ (2 × 5 m). The N (ammonium saltpeter 34.4% N) and NPK (ammonium saltpeter 34.4% N + granulated superphosphate 19% P_2_O_5_ + potassium chloride 60% K_2_O) application scheme of 9 treatments in 2 replications (*n* = 18) of semi-natural sward (>20 yrs abandoned former sown sward): control (0); N_60_; N_120_; N_180_; N_240_; N_180_P_120_; N_180_K_150_; N_60_P_40_K_50_; N_180_P_120_K_150_. Investigated cultural pasture (CP) was fertilized with N_180_P_120_K_150_ sum year rate. P and K were applied before plant vegetation in early spring, and N fertilizer was applied two times: end of April and after 1st cut (beginning of July) in all grasslands. Fresh mass (FM) weighting (g 0.2 m^−2^ per treatment, *n* = 20) and drying (105°C) were used to determine grassland productivity (g m^−2^) and obtain dry materials (DM, %). Grassland botanical composition was determined on harvested vegetation.

GHG (CO_2_, N_2_O, and CH_4_) emissions were monitored by the static chamber method [50] using opaque circular chambers (0.05 m^−3^), with 6 replicates per treatment (*n* = 60). Cylindrical steel collar (20 cm high and 43 cm diameter) was inserted into the soil to a depth of 6 cm. Two collars and chambers were placed in each treatment. The collar frames remained in the soil and were open to the atmosphere between samplings, except when removed for tillage and sowing. During the measurements, the chambers were closed with an airtight lid simultaneously in all treatments. Chamber air was sampled 3 times in one-hour interval period. Gas fluxes were measured on 4 different dates in grasslands.

 The measurements were carried out 2 or 3 weeks after fertilizer application every month between June and September in the absence of frost stress. The gas samples were analyzed in the laboratory by nondispersive infrared gas analyzer (MGA3000; ISO 9001 : 2000) calibrated separately for each gas using ML-800 gas standard (2 atm) in accordance with LST/ISO: 1401: 2005. Gas samples were analyzed on the same day evaluating volume concentrations (ppm) of trace gases. Daily net exchange (mg h^−1^ m^−2^) of CO_2_, CH_4_, and N_2_O in agroecosystem was calculated by integrating the 60-minute fluxes determined by the meteorological measurements over each day.

Thermal and irrigation conditions during vegetation period were characterized by sum of monthly precipitation (Pr) and active air temperature (*T*) (>10°C), accordingly to commonly used in Europe G. Selianinov (1928) hydrothermal coefficient (HTK) [51]. High rates of hydrothermal coefficient (HTC = 2.0 and 4.0) indicated moisture abundance in June and August, but it was optimal in July (HTC = 1.6) and too dry (HTK = 0.9) in September 2009 (Figure 1).

### 2.3. Data Analysis

Multiple-criteria decision-making (MCDM) methods enable to choose the best alternative from either finite or infinite set of alternatives. Multiple-attribute decision-making (MADM) methods are applied when dealing with the former class of problems. The term MCDM will henceforth refer to MADM methods in this paper. Noteworthy, MCDM methods can be applied when performing multidimensional analysis, as these methods evaluate the alternatives according to system of indicators rather than certain single indicator. The latter practice would lead to monocriterion analysis which may be unsuitable for some complex issues.

Roy [52] presented the following pattern of MCDM problems: (1) *α* *choosing* problematique—choosing the best alternative from a set of available alternatives; (2) *β* *sorting* problematique—classifying alternatives of a set of available alternatives into relatively homogenous groups; (3) *γ* *ranking* problematique—ranking alternatives of a set of available alternatives from best to worst; (4) *δ* *describing* problematique—describing alternatives of a set of available alternatives in terms of their peculiarities and features.

This section describes additive ratio assessment (ARAS) method as reported by Zavadskas and Turskis [44]. The ARAS method was chosen for analysis due to its effectiveness and suitability for compromise selection.

In the first stage, the multiple-criteria decision-making matrix *X* is formed. The matrix consists of *m* rows representing respective alternatives and *n* columns identifying certain criteria:

(1) $$X=\left[\begin{array}{ccccc} x_{01} & \cdots & x_{0j} & \cdots & x_{\mathrm{on}}\\ \vdots & \ddots & \vdots & ⋰ & \vdots \\ x_{i1} & \cdots & x_{ij} & \cdots & x_{\text{in}}\\ \vdots & ⋰ & \vdots & \ddots & \vdots \\ x_{m1} & \cdots & x_{mj} & \cdots & x_{mn} \end{array}\right],$$

where *i* denotes the *i*th fertilizing option, with *m* being the cardinality of fertilizing regimes. In our case, we have *m* = 10. Noteworthy, *x*_0*j*_ are the *j*th attribute (criterion) of the best ideal solution, and *n* is the number of indications considered, namely, emission (CO_2_, CH_4_, and N_2_O) and yield indices (FM, DM, 3 botanical groups). Indeed, the aforementioned indicator is commonly used in assessment of agrosector environment and productivity [29, 49, 53]. In our study, we have *n* = 60. Indeed, the values of the optimal solution, can be defined either (1) by putting in preknown optimal values of certain phenomenon or (2) by selecting the maxima of benefit criteria (on the contrary, minima for cost criteria):

(2) $$\begin{array}{c} x_{0j}=\underset{i}{\max}{\;x}_{ij},\;\forall j\in B,\\ x_{0j}=\underset{i}{\min}{\;x}_{ij},\;\forall j\in C. \end{array}$$

with *B* and *C* being the sets of benefit and cost criteria, respectively. In addition, each criterion can be assigned with the significance coefficient  *w*_*j*_, such that  ∑_*j*_ *w*_*j*_ = 1.

The second stage of evaluation encompasses normalization of the matrix *X*. As a result, a normalized decision-making matrix $\overset{®}{X}$ is formed, where its elements ${\overset{®}{x}}_{ij}$ are computed in the following way:

(3) $$\begin{array}{c} {\overset{®}{x}}_{ij}=\frac{x_{ij}}{\sum_{i=0}^{m}x_{ij}},\;\forall j\in B,\\ {\overset{®}{x}}_{ij}=\frac{1/x_{ij}}{\sum_{i=0}^{m}1/x_{ij}},\;\forall j\in C. \end{array}$$

Consequently, the responses of each alternative on objectives are transformed into dimensionless numbers which are suitable for multiple-criteria evaluation. Moreover, the normalized matrix $\overset{®}{X}$ is weighed by multiplying each element of the matrix from respective coefficient of significance

(4) $${\hat{x}}_{ij}={\overset{®}{x}}_{ij}w_{j},\;\forall i=0,1,\ldots ,m,$$

where ${\hat{x}}_{ij}$ is the weighted normalized value of the *j*th criterion for the *i*th alternative. In the last stage, the values of utility function are approximated for each of alternatives

(5) $$S_{i}=\overset{n}{\underset{j=1}{\sum }}{\hat{x}}_{ij},\;i=0,1,\ldots ,m.$$

As the ideal solution has been defined in the first stage, it is possible to compare the utility of each remaining alternative with that of the ideal solution

(6) $$K_{i}=\frac{S_{i}}{S_{0}},\;i=1,2,\ldots ,m,$$

where *K*_*i*_ is the relative utility index of the *i*th alternative. It is obvious that values of *K*_*i*_ range between 0 and 1. The best alternative therefore is chosen by maximizing  *K*_*i*_.

## 3. Results and Discussion

Multiple indices, considered during the analysis, present the complexity of fluctuating environment during experimental period in study sites. Variation of climatic and soil physical indices resembles changing background for vegetation of plant-microorganisms complex [54] as well as GHG formation in plant-soil complex of grasslands ecosystems and thus supports explanation of observed differences in GHG emissions during study period.

**Figure 2:**
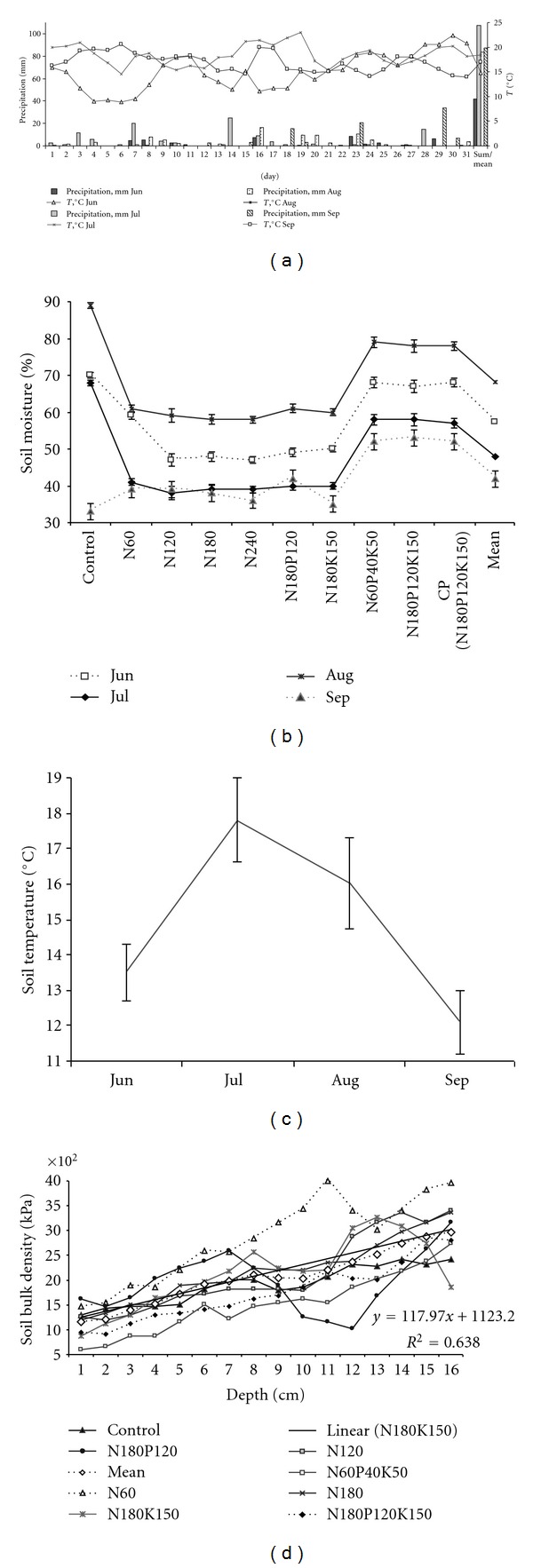
Meteorological conditions (a) and variation of soil characteristics during study period (JUN–SEP): (b) moisture, (c) temperature, and (d) soil bulk density at different depth (mean ± SE).

Flux rates of CO_2_, N_2_O, and CH_4 _ were measured during summer to avoid negative effect of spring or autumn frosts. Air and soil physical peculiarities fluctuated during study period and hence generated different conditions for GHG fluxes [8, 55, 56]. Relatively low mean temperature 14.8°C and month precipitation 42 mm were observed at the beginning of summer (Figure 2). During vegetation period, maximum mean values of air temperature 18.4°C as well as the mean monthly precipitation 107.4 mm were recorded in July. Ratio of these indices has determined rate of HTC equal to 1.6 which is optimal for plant and aerobic microorganisms vegetation. In the later summer–early autumn, these indices tended to change into draught direction, whereas environmental conditions became less suitable for vegetation of different organisms and biomass formation.

The lowest values of mean soil temperature 12.1°C, monthly sum of precipitation 28.3 mm, and mean gravimetric moisture 41.9% (Figure 1(a, b, c)) were in September and have determined the drought condition (HTC = 0.9). The most favorable environment conditions for organism vegetation and GHG emissions from agricultural soils [21, 30] were registered in July. Due to decreased air temperature (16.9°C) and soil temperature (16.0°C) as well as abundant precipitation rate 87.5 mm per month, surplus wet conditions (soil moisture 68.1% and HTC = 4.0) were observed in August. Noteworthy, redundant moisture forms anaerobic conditions in soil, thus creating unfavorable background for aerobic microbes, but nevertheless stimulating activity of anaerobic microbes and CH_4 _ production [22]. Nonetheless, only negligible increase of CH_4_ emissions was recorded in August (Table 3). 

As the degree of soil compactness similarly influences crop growth and microbes vegetation along most of soils, it can be assumed that it also similarly influences the most significant compaction-dependent growth factors [57]. The factors usually identified as the most critical in excessively compacted soils are aeration and root penetration resistance. Therefore, they are of special interest here [58]. Soil compactness was observed as directly and significantly (*r* = 0.9) dependent on depth (Figure 1(a, b, c)). Soil mean compactness in the plough layer (5–25 cm) ranged between 1158 and 2045 k Pa in sites of fertilized grassland treatment. Moreover, fertilizing rates influence soil chemical composition (Table 1). Soil pH value above 7 was recorded. N_total_ ranged within 0.90–1.45%, P_2_O_5_ was within 108.00–225.00 mg kg^−1^, and K_2_O was within 117.00–152.00 mg kg^−1^.

Increasing rates of fertilizer significantly (*r* = 0.9) induced grasslands productivity. The highest yields of FM 6045 g m^−2^ and DM 1553.6 g m^−2^ were recorded in CP treatment. The lowest yields of FM 892.5–957.5 g m^−2^ and DM 190.2–203.8 g m^−2^ were observed in control and N_60_ treatment. As recorded in [59, 60], grassland biological diversity and composition are significantly related with field management and particularly with fertilizing. This corresponded with observed changes in botanical composition by decreasing legumes (*r* = 0.3) content when N_120_ and higher rates were applied in seminatural swards (Table 2). Nonetheless, grasses (*r* = 0.8) tolerate heavier N rates, and their share increased in sward. Change in botanical composition possible induced different assimilation of fertilizers as well as emissions rates in grassland.

The obtained data indicate significant rates of CO_2_, N_2_O emissions in contradistinction to negligible rates of CH_4 _ from both seminatural grassland and cultural pasture (Table 3). Decrease of N_2_O emission during vegetation from June to September was evaluated due to changed activity producing nitrous oxide microorganisms.

Their activity depends not only on dissoluble substrate concentration [18] fertilizer rates and type [17, 61], but also on environment temperature, humidity, CO_2 _ concentration, and so forth [45, 62]. In regard with references, there was medium correlation between N_2_O emission and soil humidity (*r* = 0.5) and pH (*r* = 0.6) determined. Nonetheless, strong interaction (*R* = 0.9) was observed between climatic indices (temperature and precipitation), fertilizing, and N_2_O emission in grasslands. Nitrous oxide (N_2_O) is the main biogenic greenhouse gas contributing to the global warming potential (GWP) of agroecosystems and therefore requires a capacity to predict N_2_O emissions in relation to environmental conditions and crop management [63].

It is evident that the CO_2_ efflux from any agricultural system is the net result of autotrophic fixation and heterotrophic respiration and as such depends on the combination of environmental conditions and management practices [56]. Closely related with respiration and photosynthesis, formation of CO_2 _ impacted on environment temperature (*r* = 0.8), humidity (*r* = 0.8), soil pH (*r* = 0.7), and nutritional materials in regards with reports [23]. Produced CO_2 _ has strong correlation with fertilizing rates (*r* = 0.8) in fertilized abandoned grassland. In accordance with [2, 11, 64], CO_2 _ emission still depended heavily on monomial fertilizers rates (*r* = 0.8) in seminatural and cultural grassland.

According to IPCC [2] and Lehuger et al. [65], methane follows after CO_2 _ and is second in order of importance with 23 times higher warming effect. Nonetheless, significantly large CH_4_ content could be observed in anaerobic conditions [53]. CH_4_ formation on well-drained soils is performed by aerobic microorganisms also, but there methane is oxidized by methanotrophic and nitrifying bacteria; therefore, higher content does not accumulate in well-drained soils [66, 67]. Due to this tendency, very low CH_4 _ emission was observed in all investigated grasslands arranged in well-drained soils of training farm (Table 3). Measured CH_4 _ emission was negligible and ranged between 0.01 and 0.06 *μ*g h^−1^ m^−2^ in fertilized grassland.

As monocriteria methods cannot successfully cope with sets of indicators describing greenhouse gas (CO_2_, N_2_O, and CH_4_) emissions and thus determine the most compromise fertilizing schemes, a new multi-criteria method was employed. Multi-criteria decision-making (MCDM) by applying additive ratio assessment (ARAS) method was used for choosing the most environmentally sustainable fertilizing scheme in seminatural grassland. The multi-criteria decision matrix (Table 4) was formed according to the above-described findings. The hypothetic ideal solution was defined according to (2). As generally used in agroecosystem evaluation [26, 53], there were selected two groups of indicators, each covering three environmental and productivity indices, respectively. Hence, each indicator was attributed with equal significance coefficient of 1/6. Finally, the weighted normalized decision-making matrix (Table 5) was formed as a result of data normalization and weighing as defined by (3) and (4), respectively. 

**Figure 3:**
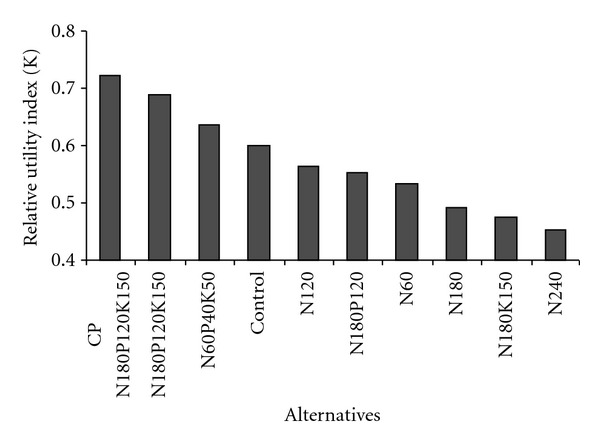
Relative utility of different treatments (comparison with the hypothetic ideal solution) in grassland agroecosystems.

The final ranks were retrieved on a basis of relative utility indicators *K*_*i*_, which were obtained by employing (5) and (6). The applied quantitative analysis assumes that studied agroecosystems' behavior can be fully grasped or satisfactorily simplified within a single figure (Figure 3). The outcome of ranking according to given data indicates the most compromise fertilizing regime. The most effective fertilizing N_180_P_120_K_150 _ was identified for cultural pasture (*K* = 0.72). This result indicates high ability of PC to assimilate hard fertilizers rates with optimal ratio of production and other evaluated environmental indices, whereas seminatural grassland fertilized with the same rate represented less efficiency and environmental conditions (*K* = 0.69) possibly due to worse assimilation peculiarities of composed species. Therefore, fertilizing with N_60_P_40_K_50_ can be suggested as the best management way (*K* = 0.64) for seminatural grassland which ensures sustainability according to evaluated environment indices.

Applied quantitative analysis assumes that studied agroecosystems' behavior can be fully grasped or satisfactorily simplified with a single figure (Figure 3).

The outcome of multiple ranking according to given data indicates the best alternative meeting the fertilizing requirements. N_180_P_120_K_150 _ was identified as the most effective fertilizing for cultural pasture (*K* = 0.72). This result indicates high ability of CP to assimilate hard fertilizers rates with optimal ratio of production and other evaluated environmental indices [68], whereas seminatural grassland fertilized with the same rate occurred to be less efficient (*K* = 0.69) possibly due to worse nutritional assimilation peculiarities of composing species and thus higher GHG emissions rate. This index decline could be explained by the change in botanical composition of sward as well. Unproductive species of forbs' botanical group has been gradually establishing in abandoned grassland, thus application of heavy rate N_180_P_120_K_150 _ is economically inefficient.

Ecological impact of N_60_P_40_K_50_ rate to protect soil from impoverishment must be noted because of link to a number of biophysical and socioeconomic factors [69]. 495.5 g m^−2^ DM yield indicated mediate inference of rate N_60_P_40_K_50_ capacity to conserved soil fertility in abandoned grassland. Moreover, this medium level of harvest might be enough for undemanding cattle (sheep or goats), thereby allowing extensive use by grazing which in turn prevents establishment of the climatic cenosis (forest) in abandoned grassland. Summarizing, fertilizing N_60_P_40_K_50_ can be stated as the best compromise management way (*K* = 0.64) for low productivity seminatural grassland which provides sustainable impact on evaluated environment and productivity indices. The application of multi-criteria decision-making method therefore enabled to choose the optimal compromise alternative for fertilizing management simultaneously considering multiple objectives, namely, mitigation of atmospheric pollution with anthropogenic GHG and maximization of grassland yield.

## 4. Conclusion

Application of new additive ratio assessment (ARAS) method facilitates the structuring of different rates of monomial nitrogen, and multiple fertilizers impacted significant drivers of anthropogenic pollution and climate change—GHG emissions—as well as other environmental indices.

Given fertilizing is easily controlled factor, fertilizing management may therefore be important when diminishing emissions in grasslands. Climatic conditions, namely, temperature and humidity, strongly (*r* = 0.9) impacted the rates of GHG emissions during vegetation. The lowest CH_4 _ emission was observed in grasslands, probably due to well-drained soil conditions. The highest GHG emission (0.045 mg h^−1^ m^−2^ N_2_O, 23.49 mg h^−1^ m^−2^ CO_2 _ and 0.06 *μ*g h^−1^ m^−2^ CH_4_) was observed on June in seminatural grassland. Nonetheless, lower emissions were observed in cultural grassland. This finding can be possible justified by the fact that species peculiar to cultural grassland exercise higher physiological potential to assimilate fertilizers when forming yield. Gradual decline of GHG fluxes was observed during vegetation, in accordance with decreasing supply of environmental components encompassing organic substrates, fertilizers, activity of microorganisms, and their interaction with humidity and temperature.

There was strong correlation observed between mean N_2_O, CO_2_, and CH_4 _ emission during vegetation period on the one hand, and NPK (*r* = 0.9, 0.8 and 0.9) with monomial nitrogen fertilizers (*r* = 0.8 and 0.6) on the other hand. Therefore, appropriate and environmentally sustainable fertilizing rate for supporting soil fertility and contributing to significant driver of climate change—anthropogenic GHG emission reduction—should not exceed N_60_P_40_K_50 _ for seminatural grassland in the Central Lithuania.

## Figures and Tables

**Table 1:** Soil agrochemical mean parameters of seminatural grassland and cultural pasture (CP) at the end of vegetation period (2009) (*P* < 0.05).

Treatment	pH	N_total_, %	P_2_O_5_, mg kg^−1^	K_2_O, mg kg^−1^
Control	7.25	1.00	132.50	146.50
N_60_	7.10	1.11	146.50	152.00
N_120_	7.15	0.93	127.00	139.00
N_180_	7.15	0.90	125.00	129.50
N_240_	7.20	0.80	108.00	122.50
N_180_P_120_	7.30	1.35	197.00	120.00
N_180_K_150_	7.45	1.04	143.00	172.50
N_60_P_40_K_50_	7.40	1.14	166.00	117.00
N_180_P_120_K_150_	7.35	1.44	225.00	126.50
CP(N_180_P_120_K_150_)	7.25	1.45	215.90	125.00

**Table 2:** Grasslands productivity and botanical composition response to applied fertilizing and farming management (FM-fresh mass; DM-dry materials).

Alternatives	FM, g m^−2^	DM, g m^−2^	DM, %	Grasses, %	Legumes, %	Forbes, %
Control	957.5	190.2	19.9	35	35	30
N_60_	892.5	203.8	22.8	50	3	47
N_120_	1002.5	235.1	23.5	75	0	25
N_180_	1150	271.4	23.6	76	0	24
N_240_	1520	292.6	22.7	70	0	30
N_180_P_120_	1700	386.2	22.7	40	50	10
N_180_K_150_	1355	342.2	25.3	80	0	20
N_60_P_40_K_50_	2127.5	495.5	23.3	35	55	10
N_180_P_120_K_150_	3020	827.7	27.4	97	0	3
CP(N_180_P_120_K_150_)	6045	1553.6	25.7	58	42	0.4
LSD05	87.55	0.54	0.6	1.04	0.53	1.18

**Table 3:** Microgas emission in fertilized and differently managed grasslands (seminatural and CP-cultural pasture) during vegetation period.

Alternatives	JUN	JUL	AUG	SEP	Mean	JUN	JUL	AUG	SEP	Mean	JUN	JUL	AUG	SEP	Mean
	CO_2_ emission, mg h^−1^ m^−2^					N_2_O emission, mg h^−1^ m^−2^					CH_4 _ emission, *μ*g h^−1^ m^−2^				
Control	3.19	3.76	1.65	0.06	2.17	0.023	0.014	0.011	0.008	0.014	0.01	0.05	0.05	0.00	0.040
N_60_	2.49	5.61	3.77	0.11	2.99	0.022	0.018	0.017	0.009	0.017	0.06	0.01	0.01	0.00	0.026
N_120_	5.59	6.23	6.97	0.18	4.74	0.025	0.024	0.019	0.010	0.019	0.03	0.01	0.01	0.00	0.015
N_180_	12.77	9.30	5.97	0.18	7.06	0.027	0.024	0.023	0.011	0.021	0.03	0.02	0.02	0.00	0.022
N_240_	10.98	14.06	8.38	0.29	8.42	0.029	0.024	0.023	0.013	0.022	0.04	0.02	0.02	0.00	0.025
N_180_P_120_	—	9.59	7.80	0.28	5.89	0.036	0.018	0.016	0.011	0.020	0.05	0.02	0.02	0.00	0.029
N_180_K_150_	12.86	12.40	8.98	0.29	8.64	0.039	0.028	0.021	0.011	0.025	0.05	0.02	0.02	0.00	0.029
N_60_P_40_K_50_	4.56	5.68	4.16	0.21	3.65	0.024	0.024	0.022	0.011	0.021	0.06	0.03	0.03	0.00	0.038
N_180_P_120_K_150_	23.49	11.21	8.97	0.48	11.04	0.045	0.018	0.009	0.010	0.020	0.04	0.03	0.03	0.00	0.032
CP(N_180_P_120_K_150_)	13.25	14.04	8.08	0.45	8.95	0.042	0.025	0.017	0.009	0.023	0.04	0.02	0.02	0.00	0.020
LSD05	0.121	0.19	0.11	0.012	0.10	0.001	0.001	0.001	0.000	0.001	0.012	0.011	0.011	0.000	0.0011

**Table 4:** Measurement results in grasslands (initial decision-making matrix *X*). FM-fresh mass; DM-dry materials.

Alternatives	FM, g m^−2^	DM, g m^−2^	Grasses, %	CH_4_ emission, %	CO_2_ emission, mg h^−1^ m^−2^	N_2_O emission, mg h^−1^ m^−2^
	(1)	(2)	(3)	(4)	(5)	(6)

*Direction of optimization*	MAX	MAX	MAX	MIN	MIN	MIN
*Weights*	0.166667	0.166667	0.166667	0.166667	0.166667	0.166667
Ideal solution	3020	827.6948	98	0.015479	2.166783	0.0141
Control	957.5	190.1977	70	0.039592	2.166783	0.0141
N_60_	892.5	203.8013	53	0.025849	2.994347	0.016642
N_120_	1002.5	235.0942	75	0.015479	4.742146	0.019484
N_180_	1150	271.4	76	0.022	7.055962	0.022147
N_240_	1520	192.5579	70	0.025442	8.424319	0.024235
N_180_P_120_	1700	386.1619	90	0.029429	5.888983	0.022218
N_180_K_150_	1355	342.1966	80	0.0293	8.635847	0.024861
N_60_P_40_K_50_	2127.5	495.4876	90	0.038453	3.652983	0.020537
N_180_P_120_K_150_	3020	827.6948	97	0.031774	11.03944	0.024359
CP(N_180_P_120_K_150_)	2786	795	98	0.021151	8.952235	0.023349

**Table 5:** Normalized values (${\hat{x}}_{ij}$) of decision-making matrix according to ARAS method.

Alternatives	(1)	(2)	(3)	(4)	(5)	(6)	*S *	*K*
Ideal Solution	0.025771	0.028937	0.018209	0.023886	0.030892	0.021203	0.148897	1
Control	0.008171	0.006649	0.013006	0.009339	0.030892	0.021203	0.08926	0.599472
N_60_	0.007616	0.007125	0.009848	0.014303	0.022354	0.017965	0.079211	0.531983
N_120_	0.008555	0.008219	0.013935	0.023886	0.014115	0.015344	0.084055	0.564514
N_180_	0.009813	0.009488	0.014121	0.016806	0.009486	0.013499	0.073215	0.491713
N_240_	0.012971	0.006732	0.013006	0.014532	0.007946	0.012336	0.067523	0.453489
N_180_P_120_	0.014507	0.0135	0.016722	0.012563	0.011366	0.013456	0.082115	0.551491
N_180_K_150_	0.011563	0.011963	0.014864	0.012619	0.007751	0.012025	0.070786	0.475401
N_60_P_40_K_50_	0.018155	0.017322	0.016722	0.009615	0.018324	0.014558	0.094696	0.635985
N_180_P_120_K_150_	0.025771	0.028937	0.018023	0.011636	0.006063	0.012273	0.102704	0.689763
CP(N_180_P_120_K_150_)	0.023774	0.027794	0.018209	0.01748	0.007477	0.012804	0.107538	0.722232
